# Environmental cleaning is effective for the eradication of severe acute respiratory syndrome coronavirus 2 (SARS-CoV-2) virus in contaminated hospital rooms: A patient from the Diamond Princess cruise ship

**DOI:** 10.1017/ice.2020.144

**Published:** 2020-04-17

**Authors:** Yosuke Hirotsu, Makoto Maejima, Masumi Nakajima, Hitoshi Mochizuki, Masao Omata

**Affiliations:** 1 Genome Analysis Center, Yamanashi Central Hospital, Kofu, Yamanashi, Japan; 2 Division of Microbiology in Clinical Laboratory, Yamanashi Central Hospital, Kofu, Yamanashi, Japan; 3 Division of Infection Disease Control, Yamanashi Central Hospital, Kofu, Yamanashi, Japan; 4 Department of Gastroenterology, Yamanashi Central Hospital, Kofu, Yamanashi, Japan; 5 The University of Tokyo, Tokyo, Japan

*To the Editor*—Doctors, nurses, and other medical staff are greatly concerned about nosocomial outbreaks of severe acute respiratory syndrome coronavirus 2 (SARS-CoV-2). Environmental contamination is a possible source of nosocomial transmission.^1,2^ However, how effective environmental cleaning is against SARS-CoV-2 remains unclear.

A 75-year-old man infected with SARS-CoV-2 was diagnosed with COVID-19 during the quarantine period on the Diamond Princess cruise ship. He was transferred directly to our hospital on February 11, 2020. He resided in patient room A for 2 days then was moved to room B, where he stayed for 19 days. After cleaning the rooms thoroughly with disinfectant (Rely^+^On Virkon, LANXESS, or RUBYSTA in Japan), we tested 15 areas that were in close contact with the patient and medical staff. Swabs were used to transfer 5 environmental samples from room A and 10 samples from room B to universal transport media (Copan, Murrieta, CA). Cleaning was conducted immediately after the patient left the rooms. Environmental sampling was conducted within 5 days and 30 min after the patient left rooms A and B, respectively. Nucleic acids were extracted using MagMAX Viral/Pathogen Nucleic Acid Isolation Kit (ThermoFisher Scientific, Waltham, MA) and were tested using real-time reverse transcription polymerase chain reaction (RT-PCR) targeting the nucleocapsid (*N*) gene of SARS-CoV-2. Seven sets of primers and probes (CDC-N1, CDC-N2, CDC-N3, YCH-N1, YCH-N2, NIID-N1, and NIID-N2) were used to detect SARS-CoV-2 as previously described (Supplemental Table 1 online).^3^ For the internal positive control, the human ribonuclease P 30 subunit (*RPP30*) gene was used. The patient’s records, timing of cleaning and sampling, and RT-PCR results were collated.

On admission, the patient had fever (39°C) and a mild cough (Supplemental Table 2 online). The chest X-ray and computed tomography scan on day 1 showed signs of pneumonia in both lungs. He received lopinavir/ritonavir and antibacterial therapy on day 2, but showed respiratory failure. He received supplemental oxygen from day 4 to day 15. After careful clinical management, the patient’s overall status improved. RT-PCR showed that his sputum was positive for SARS-CoV-2 on day 11. Subsequently, nasopharyngeal swabs were negative on days 17, 22, and 29.

The patient stayed in room A for 3 days, during which he had the SARS-CoV-2 infection. After cleaning room A, 5 environmental samples were examined by RT-PCR. All samples were negative for SARS-CoV-2 and were positive or negative for *RPP30* (Table 1).

**Table 1.** Real-Time RT-PCR Analysis of Environmental Samples

Location	RT-PCR (No. of Samples)	
**Patient room A**	SARS-CoV-2	Human *RPP30*
Light switch	Negative (0/1)	Negative (0/1)
Nurse call attached to the bed	Negative (0/1)	Positive (1/1)
Toilet door handle	Negative (0/1)	Negative (0/1)
Bed guard	Negative (0/1)	Positive (1/1)
**Anterior room A**		
Dust box	Negative (0/1)	Positive (1/1)
**Patient room B**		
Bed desk	Negative (0/1)	Negative (0/1)
Bed guard	Negative (0/1)	Negative (0/1)
Door handle	Negative (0/1)	Positive (1/1)
Dust box, room side	Negative (0/1)	Negative (0/1)
Dust box, corridor	Negative (0/1)	Positive (1/1)
Control panel on mechanical ventilation	Negative (0/1)	Positive (1/1)
Light switch	Negative (0/1)	Negative (0/1)
Nurse call	Negative (0/1)	Positive (1/1)
Hand soap dispenser	Negative (0/1)	Negative (0/1)
**Anterior room B**		
Sink, external rim and internal bowl	Negative (0/1)	Positive (1/1)

Note. PCR, polymerase chain reaction; RPP30, ribonuclease P 30 subunit; SARS-CoV-2, severe acute respiratory syndrome coronavirus 2.

After the patient left room A, he resided in room B for 20 days. Ten environmental samples were collected after cleaning. All 10 samples from room B were negative for SARS-CoV-2 and were positive or negative for *RPP30* (Table 1).

SARS-CoV-2 is detectable in several types of clinical samples including bronchial lavage fluid, nasopharyngeal swab, pharyngeal swab, sputum, saliva, and feces.^4,5^ Transmission of SARS-CoV-2 via surfaces in hospitals is of great concern to medical staff and patients. Blocking the potential routes of transmission is essential for preventing the spread of SARS-CoV-2.^6^ A recent study showed that environmental contamination can occur via contact with patients with SARS-CoV-2 and upper respiratory tract symptoms.^7^ After cleaning, all areas were negative for SARS-CoV-2; therefore, thorough cleaning is sufficient for SARS-CoV-2 decontamination.

This study had several limitations. First, RT-PCR was not performed before cleaning because of the risk of nosocomial transmission. Therefore, a comparison of the viral loads of high-touch areas before and after cleaning is required. Second, this study involved a single patient, and further studies are required to confirm these findings.

In summary, our data indicate the effectiveness of environmental cleaning for SARS-CoV-2 decontamination. This information is useful for infection control strategies and may alleviate the concerns of medical staff.

## Supplementary material

For supplementary material accompanying this paper visit http://dx.doi.org/10.1017/ice.2020.144. click here to view supplementary material
