# Supplementary material for: Environmental cleaning is effective for the eradication of severe acute respiratory syndrome coronavirus 2 (SARS-CoV-2) virus in contaminated hospital rooms: A patient from the Diamond Princess cruise ship
Source: Infect Control Hosp Epidemiol. 2020 Apr 17:1–2. doi: 10.1017/ice.2020.144 (PMC7308617; doi:10.1017/ice.2020.144)
Supplement: Supplementary file 1 [file S0899823X20001440sup.zip › S0899823X20001440sup001.docx]

| **Date** | **Symptoms** | **RT-PCR of SARS-CoV-2** | **Description** |
| --- | --- | --- | --- |
| Before admission |  | Positive | Patient traveled on Diamond Princess cruise ship. |
| Day 1 | Fever, mild cough, and pneumonia |  | Patient was admitted to the hospital and entered patient Room A. |
| Day 2 | Respiratory failure | |  |
| Day 3 |  |  | Patient left Room A and entered Room B. |
| Day 4 |  |  | Patient received supplemental oxygen in Room B. Room A was cleaned. |
| Day 8 |  |  | Five environmental samples were collected from Room A via swabbing and analyzed by RT-PCR. |
| Day 11 |  | Positive in sputum | |
| Day 17 |  | Negative in nasopharyngeal swab |  |
| Day 21 |  |  | Patient left Room B. Room B was cleaned. Ten environmental samples were collected from Room B via swabbing and analyzed by RT-PCR. |
| Day 22 |  | Negative in nasopharyngeal swab |  |
| Day 29 |  | Negative in nasopharyngeal swab |  |
| Day 35 | Overall status had improved |  |  |

**Supplemental Table 2. Time course of patient symptoms, environmental cleaning, and sampling**

SARS-CoV-2, severe acute respiratory syndrome coronavirus 2; RT-PCR, reverse transcriptase PCR
