# Supplementary material for: Environmental cleaning is effective for the eradication of severe acute respiratory syndrome coronavirus 2 (SARS-CoV-2) virus in contaminated hospital rooms: A patient from the Diamond Princess cruise ship
Source: Infect Control Hosp Epidemiol. 2020 Apr 17:1–2. doi: 10.1017/ice.2020.144 (PMC7308617; doi:10.1017/ice.2020.144)
Supplement: Supplementary file 1 [file S0899823X20001440sup.zip › S0899823X20001440sup002.docx]

| Supplemental Table 1. Sequences of the primers and probes used in the CDC, NIID, and YCH assays | | | | |
| --- | --- | --- | --- | --- |
| Institute | Primer name | Description | Oligonucleotide Sequence (5’>3’) | Modification |
| CDC | CDC-N1-F | 2019-nCoV_N1 Forward Primer | 5’-GACCCCAAAATCAGCGAAAT-3’ | None |
| CDC | CDC-N1-R | 2019-nCoV_N1 Reverse Primer | 5’-TCTGGTTACTGCCAGTTGAATCTG-3’ | None |
| CDC | CDC-N1-P | 2019-nCoV_N1 Probe | 5’-FAM-ACCCCGCATTACGTTTGGTGGACC-BHQ1-3’ | FAM/BHQ1 |
| CDC | CDC-N2-F | 2019-nCoV_N2 Forward Primer | 5’-TTACAAACATTGGCCGCAAA-3’ | None |
| CDC | CDC-N2-R | 2019-nCoV_N2 Reverse Primer | 5’-GCGCGACATTCCGAAGAA-3’ | None |
| CDC | CDC-N2-P | 2019-nCoV_N2 Probe | 5’-FAM-ACAATTTGCCCCCAGCGCTTCAG-BHQ1-3’ | FAM/BHQ1 |
| CDC | CDC-N3-F | 2019-nCoV_N3 Forward Primer | 5’-GGGAGCCTTGAATACACCAAAA-3’ | None |
| CDC | CDC-N3-R | 2019-nCoV_N3 Reverse Primer | 5’-TGTAGCACGATTGCAGCATTG-3’ | None |
| CDC | CDC-N3-P | 2019-nCoV_N3 Probe | 5’-FAM-AYCACATTGGCACCCGCAATCCTG-BHQ1-3’ | FAM/BHQ1 |
| CDC | RP-F | RNAse P Forward Primer | 5’-AGATTTGGACCTGCGAGCG-3’ | None |
| CDC | RP-R | RNAse P Reverse Primer | 5’-GAGCGGCTGTCTCCACAAGT-3’ | None |
| CDC | RP-P | RNAse P Probe | 5’-FAM–TTCTGACCTGAAGGCTCTGCGCG–BHQ1-3’ | FAM/BHQ1 |
| NIID | NIID-N1-F | N_Sarbeco_Forward Primer | 5’-CACATTGGCACCCGCAATC-3' | None |
| NIID | NIID-N1-R | N_Sarbeco_Reverse Primer | 5’-GAGGAACGAGAAGAGGCTTG-3' | None |
| NIID | NIID-N1-P | N_Sarbeco_Probe | 5’-FAM-ACTTCCTCAAGGAACAACATTGCCA-TAMRA-3' | FAM/TAMRA |
| NIID | NIID-N2-F | NIID_2019-nCOV_N_Forward Primer | 5’-AAATTTTGGGGACCAGGAAC-3' | None |
| NIID | NIID-N2-R | NIID_2019-nCOV_N_Reverse Primer | 5’-TGGCAGCTGTGTAGGTCAAC-3' | None |
| NIID | NIID-N2-P | NIID_2019-nCOV_N_Probe | 5’-FAM-ATGTCGCGCATTGGCATGGA-TAMRA-3' | FAM/TAMRA |
| YCH | YCH-N1-F | YCH_N1 Forward Primer | 5’-CACATTGGCACCCGCAATC-3' | None |
| YCH | YCH-N1-R | YCH_N1 Reverse Primer | 5’-GAGGAACGAGAAGAGGCTTG-3' | None |
| YCH | YCH-N1-P | YCH_N1 Probe | 5’-FAM/ACTTCCTCA/ZEN/AGGAACAACATTGCCA-IBFQ-3' | FAM/ZEN/IBFQ |
| YCH | YCH-N2-F | YCH_N1 Forward Primer | 5’-AAATTTTGGGGACCAGGAAC-3' | None |
| YCH | YCH-N2-R | YCH_N1 Reverse Primer | 5’-TGGCAGCTGTGTAGGTCAAC-3' | None |
| YCH | YCH-N2-P | YCH_N1 Probe | 5’-FAM/ATGTCGCGC/ZEN/ATTGGCATGGA-IBFQ-3' | FAM/ZEN/IBFQ |
| NIID, National Institute of Infectious Diseases; YCH, Yamanashi Central Hospital; CDC, Centers for Disease Control and Prevention | | | |  |
| FAM, 6-carboxyfluorescein; BHQ1, Black Hole Quencher 1; IBFQ, Iowa Black Fluorescent Quencher | | | |  |
